# Analyzing Differentially Expressed Genes and Pathways Associated with Pistil Abortion in Japanese Apricot via RNA-Seq

**DOI:** 10.3390/genes11091079

**Published:** 2020-09-15

**Authors:** Ting Shi, Shahid Iqbal, Aliya Ayaz, Yang Bai, Zhenpeng Pan, Xiaopeng Ni, Faisal Hayat, Muhammad Saqib Bilal, Muhammad Khuram Razzaq, Zhihong Gao

**Affiliations:** 1 Laboratory of Fruit Tree Biotechnology, College of Horticulture, Nanjing Agricultural University, Nanjing 210095, China; shiting@njau.edu.cn (T.S.); 2017204045@njau.edu.cn (S.I.); 2019204009@njau.edu.cn (Y.B.); 2017104013@njau.edu.cn (Z.P.); 2017204013@njau.edu.cn (X.N.); maken_faisal@yahoo.com (F.H.); 2 Jiangsu Key Laboratory for Horticultural Crop Genetic Improvement, No. 50 Zhongling Street, Nanjing 210014, China; 3 State Key Laboratory of Crop Genetics and Germplasm Enhancement, Ministry of Science and Technology, College of Horticulture, Nanjing Agricultural University, Nanjing 210095, China; 2018204051@njau.edu.cn; 4 Key Laboratory of Integrated Management of Crop Diseases and Pests, College of Plant Protection, Nanjing Agricultural University, Nanjing 210095, China; 2018202066@njau.edu.cn; 5 Soybean Research Institute, National Centre for Soybean Improvement, Nanjing Agricultural University, Nanjing 210095, China; 2017201098@njau.edu.cn

**Keywords:** pistil abortion, Japanese apricot, RNA-Seq, hormone signaling, metabolic pathways

## Abstract

Reproduction is a critical stage in the flower development process, and its failure causes serious problems affecting fruit quality and yield. Pistil abortion is one of the main factors in unsuccessful reproduction and occurs in many fruit plants. In Japanese apricot, the problem of pistil abortion is very common and affects fruit quality and plant yield; however, its molecular mechanism is not clearly understood. Therefore, in the current study, we used RNA-Seq to identify the differentially expressed genes (DEGs) and pathways actively involved in pistil abortion. A total of 3882 differentially expressed genes were found after cutoff and pairwise comparison analysis. According to KEGG pathway analysis, plant hormone signaling transduction and metabolic pathways were found most significantly enriched in this study. A total of 60 transcription factor families such as MADS-box, NAC and TCP showed their role in this process. RT-qPCR assays confirmed that the expression levels were consistent with RNA-Seq results. This study provides an alternative to be considered for further studies and understanding of pistil abortion processes in Japanese apricot, and it provides a reference related to this issue for other deciduous fruit crops.

## 1. Introduction

Japanese apricot (*Prunus mume* Sieb. et Zucc) is an important fruit and ornamental plant. It has great economic importance, high profit and world market demand, and it is used in value-added products like jams, pickles, etc. [1,2]. Flower development is a fundamental stage in the life cycle of the plant and plays a significant role in the sexual reproduction process [3]. Pistil abortion is a widespread phenomenon occurring in fruit plants and has been discussed in different fruit crops like pomegranate, *Xanthoceras sorbifolia* and olive [4,5,6]. In Japanese apricot, the problem of pistil abortion is very common and causes serious losses, such as decrease in fruit quality and yield, thus limiting the apricot growing industry. The abortive pistils are characterized as withered or absent (no pistils) [7]. Several proteomic studies have been performed and found that glucose, starch and photosynthesis metabolisms were associated with pistil abortion [8].

In fruit trees, pistil abortion may result from improper ovary development, style formation and fertilization during flower development [9]. The rate of pistil abortion depends on the type of apricot cultivar, as the abortion rate in Japanese apricot fruit can reach up to 76.3% [10], while in some cultivars of Xinjiang apricot the pistil abortion rate can range from 60.4% to 99.64% [11]. Numerous studies showed that pistil abortion might be controlled through many factors such as the environment (light, humidity, temperature), endogenous substances and signals associated with various biological and cellular processes during floral organ formation [12].

Plant hormones are actively involved in floral transition and floral organ development [13], and they play a regulatory role in physiological processes of flower bud development including floral bud initiation [14], differentiation [15] and dormancy [16]. As an excellent growth regulator, abscisic acid (ABA) is an active hormone involved in floral time transition [17]. In *Arabidopsis*, cytokinin (CK) promotes flowering [18,19], while gibberellin (GA) promotes cell division and growth [20]. Cytokinin affects the expression of *PIN1* protein through promoting the expression of *AG* and *SPL* genes, thereby affecting normal pistil and ovule development [21]. Therefore, the above related information confirms the substantial involvement of plant hormones during pistil abortion.

RNA-Seq technology is extensively used to determine gene expression and the associated genetic network during flower development [22], and it has previously been used in studies of sugar apple [23], pomegranate [4] and olive [24]. The problem of pistil abortion in Japanese apricot is very common but has not been well studied. Therefore, in the current study, we used RNA-Seq to investigate the molecular mechanism underlying pistil abortion. This study provides a theoretical basis for stimulating flower and pistil development and has practical significance for improving yield and quality of Japanese apricot.

## 2. Materials and Methods

### 2.1. Experimental Material

The flower buds of Japanese apricot (*P. mume*) cultivars ‘Longyan (LY)’ and ‘Daqiandi (DQD)’ were collected before opening for this experiment. The pistils were excised from the flower buds. The cv. Longyan pistils were used as normal pistils (NPs) and cv. Daqiandi pistils were used as abortive pistils (APs). All samples were collected from Japanese apricot plants grown for 8 years at the National Field Gene-bank for apricot in Nanjing, Jiangsu, China (E 119°11′12”, N 31°35′11”) from the end of November to mid-February. The plants were grown in a temperate climate with an average annual precipitation of 1106 mm and annual average temperature of 15.6 °C. The collected samples were instantly ice-covered and placed in liquid nitrogen and then stored at −80 °C until further experiments.

### 2.2. RNA Extraction and cDNA Library Preparation

The RNA was extracted by the Foregene Nucleic Acid Extraction Kit (Shenzhen, China) following the manufacturer’s standard protocol and purified using RNase-free DNase I (TaKaRa, Shiga, Japan). The RNA concentration was checked using a 2200 Bioanalyzer (Agilent Technologies, Inc., Santa Clara, CA, USA), and the quality was determined through gel electrophoresis. Then, the RNA was treated with oligo (dT) and combined with fragment buffer to synthesize cDNA. After purification, the fragment was isolated in EB end repair and 1-A nucleotide addition. Then, the ligation product was amplified through PCR, and Illumina sequencing HiSeq 4000 (BGI, Beijing, China) was used for pair-end sequencing. An overview of samples, experimental route and analysis is shown in Figure S1.

### 2.3. Quality Control and Reads Mapping

The raw reads (FASTQ) were filtered to remove the low-quality reads and unknown base N content using SOAPnuke (v 1.4.0) software. After obtaining clean reads, HISAT (Hierarchical Indexing for Spliced Alignment of Transcripts) (v 2.1.0) [25] was used to compare the clean reads to the *P. mume* genome available at (https://www.ncbi.nlm.nih.gov/genome/?term=prunus%20mume). Bowtie2 (v 2.2.5) software [26] (–q—phred64—sensitive—dpad 0—gbar 99999999–mp 1,1—np 1—score-min L,0,-0.1–p 16–k 200) was used to compare the clean reads to genomic sequences, and the gene expression level was calculated by RSEM (default parameter) (v 1.2.8).

### 2.4. RNA-Seq Data Analysis

After sequencing, the expression level of each sequence library was standardized as FPKM (Fragments Per Kilobase of Transcript per Million), and the most differentially expressed genes (DEGs) were selected for further analysis. Hierarchical clustering was performed using TBtools (v 0.667). The significance level of the transcripts was measured using the FDR (False Discovery Rate) control method [27] to rationalize *p*-values. Gene regulation was determined through cutoff values with an absolute log_2_ fold change of ≥+1 (up-regulated) and ≤−1 (down-regulated) method with *p*-values less than 0.001.

To find out the putative transcription factor (TF) related to these DEGs, Getorf (EMBOSS: 6.5.7.0) was used to identify ORF (Open Reading Frame), hmmsearch (v 3.0) was used to align ORF to the TF protein domain, and then the unigenes were identified according to TF family characterized by PlantTFDB 4.0. To further characterize the function of DEGs, GO (Gene Ontology) and KEGG (Kyoto Encyclopedia of Genes and Genomes) enrichment were performed. Based on DEGs, GO is functionally categorized as follows: (1) biological process (BP), (2) cellular component (CC) and (3) molecular function (MF). On the basis of differential gene expression analysis, the biological functions of differentially expressed genes were further understood by using GO (Gene Ontology). GO functional enrichment analysis was carried out according to their functional classes using FDR ≤0.05 as the significant enrichment level. For pathway analysis, all identified DEGs were mapped to the KEGG database. Enrichment analysis of both GO and KEGG was performed using the phyper function in R software with *p*-value < 0.05 [28]. The data from sequencing were deposited to SRA-NCBI under accession PRJNA646597.

### 2.5. Endogenous Hormone Measurement

The phytohormone contents were determined using the HPLC method described earlier [29]. The samples identical to RNA-Seq were used for hormone measurements. Three biological replications were used for each sample.

### 2.6. Quantitative Reverse Transcription PCR for Data Validation

Twelve different genes were randomly selected to verify the RNA-Seq results. Gene sequences were extracted, and their primers were designed using Primer 3 software. RNA extraction and quality tests were performed as described above, and RT-qPCR was performed according to the method described by [30,31]. Using RPII as a reference internal gene, the expression levels of the genes were analyzed via the 2^−△△CT^ method [32].

## 3. Results

### 3.1. An Overview of RNA-Seq Libraries

In the current study, six different cDNA libraries were constructed for normal and abortive pistils and sequenced using Illumina sequencing, producing an average of 65.8 M raw reads per sample. After the removal of raw reads and adopter sequences, 63.27 M clean reads per sample were obtained and mapped to the *P. mume* genome. After mapping to the reference genome, a total of 86.64% mapped reads were obtained from each sample, while uniquely mapped reads were 63.94% per sample (Table 1).

### 3.2. DEG Analysis of Normal and Abortive Pistils

**Figure 1:**
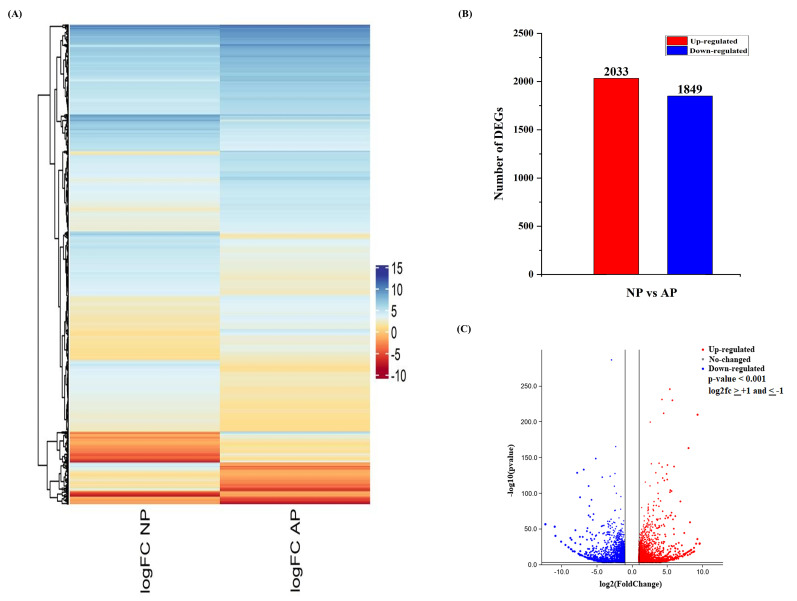
An overview of differentially expressed genes (DEGs) involved in normal and abortive pistils of Japanese apricot. (**A**) Heatmap showing the differential expression pattern of the DEGs. The color scale shows the gene expression values (log2fc). (**B**) Up- and down-regulation of differentially expressed genes. (**C**) Volcano plot showing the differentially expressed genes. The x-axis represents the log2 fold change conversion of the values, and the y-axis represents the significance value after –log10 conversion. Red shows up-regulated DEGs, blue shows down-regulated DEGs, while grey represents no DEGs.

Hierarchical clustering was performed for differentially expressed genes on the basis of their fold change values (log_2_fc), and the clustering is shown in Figure 1A. Pairwise comparison was used to analyze the regulation (up/down) and gene expression profiles between AP and NP. A total of 3882 unigenes (2033 up-regulated and 1849 down-regulated) were identified from normal and abortive pistil comparisons (Figure 1B). The volcano plot shows the expression level and overall distribution of differentially expressed genes between these two groups (Figure 1C). All identified DEGs and their expression values are listed in File S1.

### 3.3. DEG Functional Enrichment Analysis

**Figure 2:**
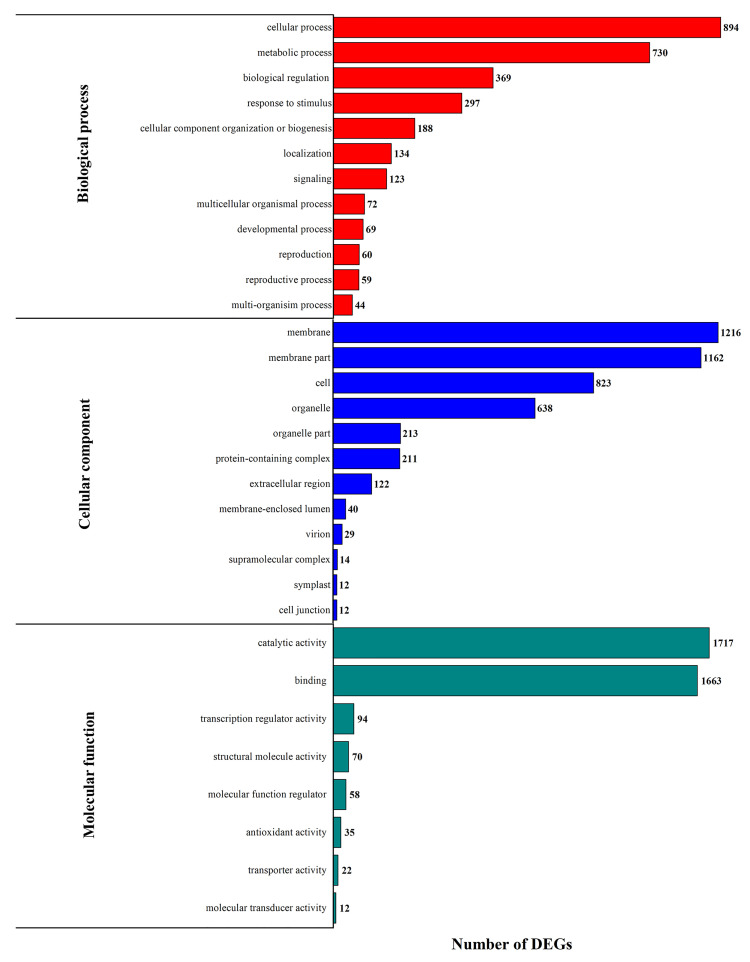
Gene Ontology (GO) analysis between normal pistil (NP) and abortive pistil (AP) showing the abundance of differentially expressed enriched GO terms.

The DEGs in NP vs AP comparisons and their functional classes were collected, and the most significant enriched terms are shown in Figure 2. In the BP category, cellular process, metabolic process and biological regulation were the leading terms; for the CC category, the most enriched terms were membrane, membrane part and cell; while in the MF category, binding and catalytic activity were the prominent terms.

**Figure 3:**
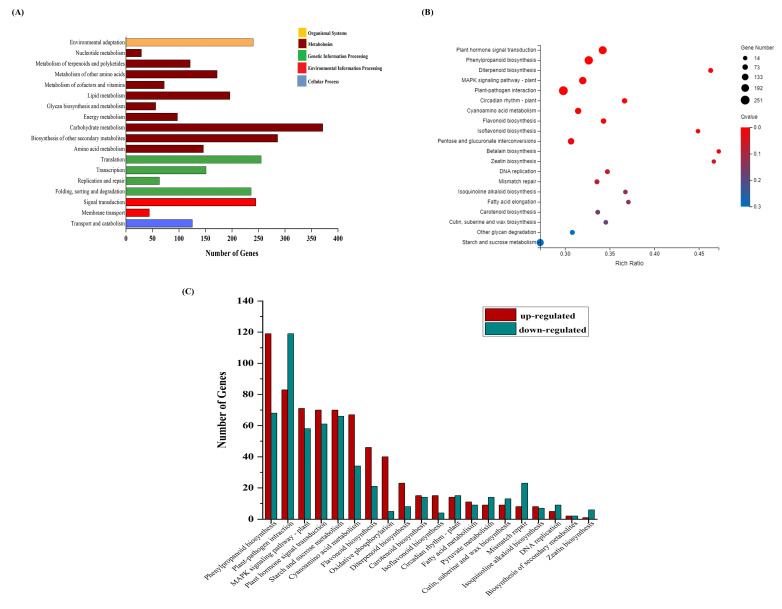
KEGG pathway enrichment analysis (**A**) DEGs related to significant enrichment analysis were involved in different processes (**B**) Bubble plot showing top20 most enriched pathways (**C**) Regulation (up/down) trend of the DEGs involved in different pathways.

For KEGG pathway study, all identified DEGs were mapped to the KEGG database, and further involvement of genes in different pathways was predicted. A total of 134 KEGG pathways were identified based on enrichment analysis (File S2). Plant hormone signal transduction, plant–pathogen interaction and different metabolic pathways were found most enriched related to this study. Gene regulation (up and down) and different processes involved in the pathway study are shown in Figure 3.

### 3.4. Expression Profiling of Transcription Factor Encoding Genes

**Figure 4:**
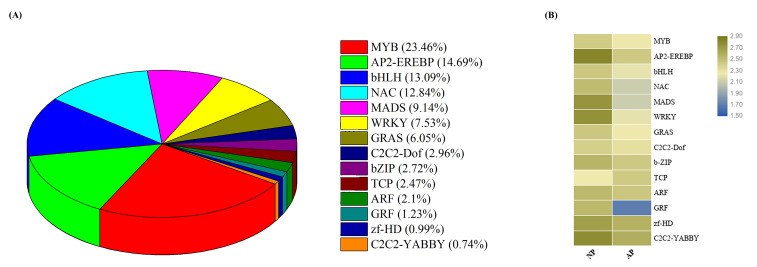
Analysis of transcription factors (TFs) between normal and abortive pistils of Japanese apricot. (**A**) Distribution of important TF families and their percentage of genes in each TF family. (**B**) Average expression profiles of genes involved in each TF family in normal and abortive pistils. The bar represents the scale of expression of each gene as indicated by blue (lower expression) and dark yellow (higher expression).

Transcription factors (TFs) are the key regulatory proteins involved in different biological and physiological processes, especially in flower organogenesis and floral development [33,34]. Therefore, in the present study, we mapped all DEGs to the plant transcription factor database (PlantTFDB) to study the expression pattern of key transcription factors. A total of 1470 TF genes were identified and divided into different transcription factor families. Among all TF families, MYB, AP2-EREBP, bHLH, NAC, MADS WRKY and TCP were the most relevant to this study. Figure 4A shows the distribution and percentage of genes in each transcription factor family, and Figure 4B shows the average expressions of genes involved in each TF family.

### 3.5. DEGs in Response to Plant Hormone Signaling Transduction Pathway

**Figure 5:**
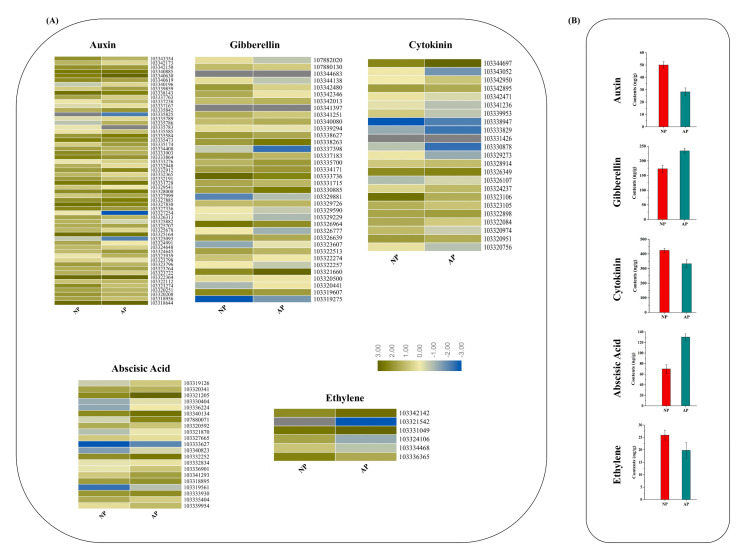
Expression profiles of the DEGs involved in plant hormones and signaling transduction pathway (**A**) Heatmap shows the expression of DEGs in normal and abortive pistils associated with different hormones like auxin, gibberellin, cytokinin, abscisic acid and ethylene. The bar represents the expression level of each gene as indicated by blue (lower expression) and dark yellow (higher expression). (**B**) Endogenous hormone contents between normal and abortive pistils. Three independent samples were collected for hormone measurement.

Earlier studies reported that various phytohormones such as auxin, cytokinin (CK), gibberellin (GA), etc., participate in different floral development processes [35]. Therefore, the regulation and expression pattern of the DEGs related to plant hormones were analyzed. An overview of the plant hormone signaling transduction pathway is shown in Figure 5 and their values are listed in File S3. In total, 59 genes in the auxin-signaling pathway, 23 genes in the cytokinin-signaling pathway, 36 genes in the GA-signaling pathway, 21 genes in the ABA-signaling pathway, 9 genes in the Ethylene-signaling pathway, 36 genes in the BR-signaling pathway, 28 genes in the JA-signaling pathway and 12 genes in the SA-signaling pathway were identified. Gene expressions related to different plant hormones involved in this study are shown in Figure 5A.

The endogenous contents of these hormones were determined in both normal and abortive pistils (Figure 5B). The contents of auxin, cytokinin and ethylene were significantly higher in normal pistils—50.03, 424.43 and 25.87 ng/g, respectively—than those of abortive pistils. The contents of gibberellin and abscisic acid were significantly higher in abortive pistils, 234.05 and 130.28 ng/g, respectively, than those of abortive pistils. Lower contents of auxin, cytokinin and ethylene—28.29, 332.78 and 19.73 ng/g—were found in abortive pistils, while lower contents of gibberellin and abscisic acid, recorded as 172.87 and 69.81 ng/g, respectively, were found in normal pistils.

### 3.6. DEGs in Response to Different Metabolic Pathways

In the present study, different metabolic pathways, such as phenylpropanoid biosynthesis (268 genes), diterpenoid biosynthesis (38 genes), flavonoid biosynthesis (90 genes), isoflavonoid biosynthesis (24 genes) and zeatin biosynthesis (15 genes), were identified. The expression patterns of DEGs involved in different biosynthesis-related pathways of normal and abortive Japanese apricot pistils were analyzed (Figure S2 and File S4).

### 3.7. Validation of Genes Through RT-qPCR

**Figure 6:**
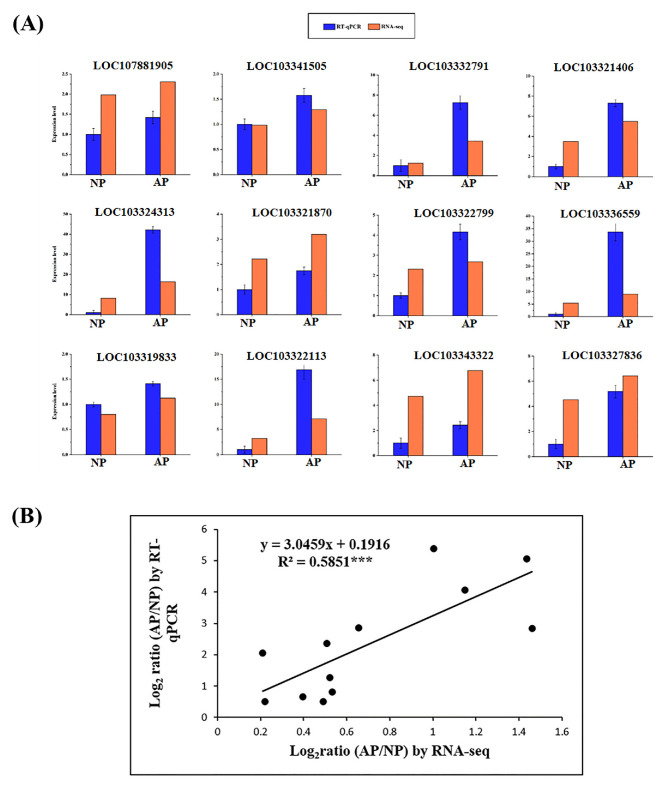
Validation of RNA-Seq data. (**A**) Verification of the expression level of the selected DEGs from RNA-Seq data through RT-qPCR. Error bars indicate the standard error as mean + SD. The x-axis represents the relative expression level, and the y-axis represents NP (normal pistils) and AP (abortive pistils). (**B**) Correlation coefficient of gene expression obtained from RT-qPCR analysis. *** shows highly significant level of the data.

To verify the results of RNA-Seq, twelve genes were randomly selected, and their relative expression level was verified through RT-qPCR. A detailed description of the selected genes and primers is listed in File S5. The expression levels of all genes were consistent with the results of RNA-Seq (Figure 6).

## 4. Discussion

Sequential regulation of genes plays a significant role in plant growth and development, and it is important to have detailed information on a gene to understand the molecular mechanism underlying any developmental process [36]. In Japanese apricot, the problem of pistil abortion also occurs. The molecular mechanism of pistil abortion in Japanese apricot is not clear. Therefore, this study aimed to analyze the regulation of genes involved in this process. Numerous studies have already reported on genome-wide transcriptional programs during reproductive development [37,38]. In the present study, RNA-Seq was performed using Illumina sequencing, and a total of 3882 genes were generated after cutoff. Cluster analysis of the identified genes was performed to uncover the expression pattern and changes during pistil abortion. Furthermore, a large number of TF families were also recognized, and some of them were strongly related to the stated problem. In general, our study provides an alternative way to further study and understand the expression profiles of molecular mechanisms and their gene regulation during pistil abortion in Japanese apricot.

TFs play an important role in flower development and regulation processes [39]. In this study, we found a large number of TF families, and most of them were closely related to flower development. From these TF families, MYB, AP2-EREBP, bHLH, NAC, MADS, WRKY and bZIP were highly expressed and have been previously reported in various plant species during the reproductive process [37]. In *Arabidopsis thaliana*, the bHLH TF family is involved in various flower development processes [40], while bZIP has also been identified in many plant species as the key regulator in flower formation and development [41]. Some other TFs such as YABBY and GRF have also been reported in many studies to have an important role in the reproductive process, and studies have shown that decreasing the expression level of GRF TFs has an effect on normal pistil development. In our study, we also noted a decreased level of GRF TFs, confirming a previous study on *Arabidopsis* [42] that this TF might have a specific role in pistil abortion in Japanese apricot.

Plant hormones are signaling molecules that play an important role in plant physiological processes such as plant metabolism, morphogenesis and growth [43], and numerous studies have shown that these hormones regulate the pistil development process [44]. In the current study, a total of 131 DEGs related to plant hormones were identified, and their expression patterns were analyzed. Auxin is an important plant hormone regulating plant growth and development [45], and the DEGs involved in this pathway were associated with AUX1, TIR1, ARF, GH3 and SAUR sub pathways. In *Arabidopsis*, ARF6 and ARF8 are highly expressed in the sepals, petals and stamen and promote floral transition [46]. In this study, most of the DEGs related to AUX/IAA, GH3, SAUR, TIR1 and ARF were up-regulated and considered to promote cell expansion, plant growth and development, which is consistent with a previous study [47]. At the same time, the content of auxin was significantly higher in normal pistils as compared to abortive pistils. Thus, auxin and its transport may be required for proper pistil development. In pistils, cytokinin is an important hormone, and an increase in CK content can increase pistil morphology and ovule numbers [48]. In this study, the content of cytokinin was significantly higher in normal pistils than abortive pistils. In the CK metabolic pathway, the genes related to *A-ARR* were down-regulated in abortive pistils, which might be a positive regulatory response to decrease the CK content in pistils, and are considered one of the most important causes for pistil abortion. In addition, GA_3_ plays an important role in flower development [49], regulates sex differentiation in plants and inhibits pistil development at an appropriate level [49]. Our results showed that the content of gibberellin was significantly higher in abortive pistils than that of normal pistils. Earlier reports in *Arabidopsis* showed that gal-3, a GA-deficient mutant, restricted floral organ growth, and its phenotype could be rescued through exogenous application of GA [50]. In the current study, GID1 (GA metabolic pathway) related genes were highly expressed, and it might be said that it reduces the biological activity of GA.

ABA is also an important hormone due to its significant role in plant growth and development, including flower development, seed development and environmental stress responses [51]. In *Arabidopsis*, ABA promotes flower bud formation through regulating flowering and photoperiod responsive genes [52], while in apples, ABA inhibits flower formation [53]. In our study, genes related to the ABA metabolic pathway showed significant expression changes, and genes related to sub pathways (ABF, PP2C and SnRK2) reported an active role during abiotic stress conditions and could adopt to any adverse environment [54,55]. In this study, the content of ABA was significantly higher in abortive pistils than in normal ones, and the genes related to ABA also showed higher expression in abortive pistils as compared to normal pistils. Ethylene is also an important hormone for floral organ development, especially pistil development, and in tobacco ethylene is an upstream regulator for ovule development [56]. In this study, the content of ethylene was significantly higher in normal pistils as compared to abortive pistils. The expression level of ethylene-related genes was also higher in normal pistils, indicating that ethylene might be a key regulator involved in pistil abortion in Japanese apricot.

Moreover, genes regulating different metabolic pathways, including phenylpropanoid biosynthesis, flavonoid and isoflavonoid biosynthesis, zeatin biosynthesis and their intermediates, had lower expression levels in abortive pistils, indicating that they were involved in the developmental process. In rice, most metabolic pathways are found enriched during floral organ development [37], as with many other plant species [57,58]. Flavonoid synthesis is required for pollen function and is very common in several floral organs [59].

## 5. Conclusions

In the current study, RNA-Seq was used to determine the regulation and expression pattern of genes in normal and abortive pistils of Japanese apricot. A total of 3882 DEGs were identified, and a comparison analysis was performed between normal and abortive pistils. We mainly focused on the expression pattern/trends of genes related to plant hormones and metabolic processes. Changes in the activity of hormones such as auxin, CK, GA and ethylene play an important role in pistil abortion. Related genes are involved in hormone synthesis and expression to regulate hormone content and promote pistil abortion under adverse conditions. Our study provides a basis for understanding the molecular mechanism of pistil abortion, and it provides a reference for future studies related to this issue in other fruit crops.

## Figures and Tables

**Table 1:** An overview of sequencing assembly in normal and abortive pistils of Japanese apricot.

Sample Name	Total Raw Reads (Mb)	Total Clean Reads (Mb)	Clean Reads Q20 (%)	Clean Reads Q30 (%)	Clean Reads Ratio (%)	Total Mapping (%)	Uniquely Mapping (%)	Accession Number
NP-1	62.47	61.66	97.21	92.63	98.71	86.19	61.90	SRR12234382
NP-2	62.52	61.72	97.22	92.67	98.80	84.95	61.07	SRR12234384
NP-3	62.47	61.74	97.12	92.41	98.83	88.24	62.97	SRR12234380
AP-1	69.96	65.16	97.48	89.81	93.13	86.42	65.53	SRR12234385
AP-2	67.47	63.54	97.59	90.16	94.18	87.77	66.69	SRR12234383
AP-3	69.96	65.83	97.61	90.22	94.08	86.28	65.53	SRR12234381

## Supplementary Materials

The following are available online at https://www.mdpi.com/2073-4425/11/9/1079/s1, Figure S1: An overview of the present study (A) The overall experimental design process (B) The route map and analysis for RNA-Seq data, Figure S2: Expression analysis of the DEGs related to different metabolic pathways involved in this study, File S1: Total number of differentially expressed genes in this study, File S2: Significantly enriched pathways and their DEGs identified in this study, File S3: Number of DEGs and their expression involved in the plant hormone signaling transduction pathway, File S4: Number of DEGs and their expression involved in different metabolic pathways, File S5: Primers used for RT-qPCR.
