# Supplementary figures and images for: Analyzing Differentially Expressed Genes and Pathways Associated with Pistil Abortion in Japanese Apricot via RNA-Seq
Source: Genes (Basel). 2020 Sep 15;11(9):1079. doi: 10.3390/genes11091079 (PMC7565994; doi:10.3390/genes11091079)

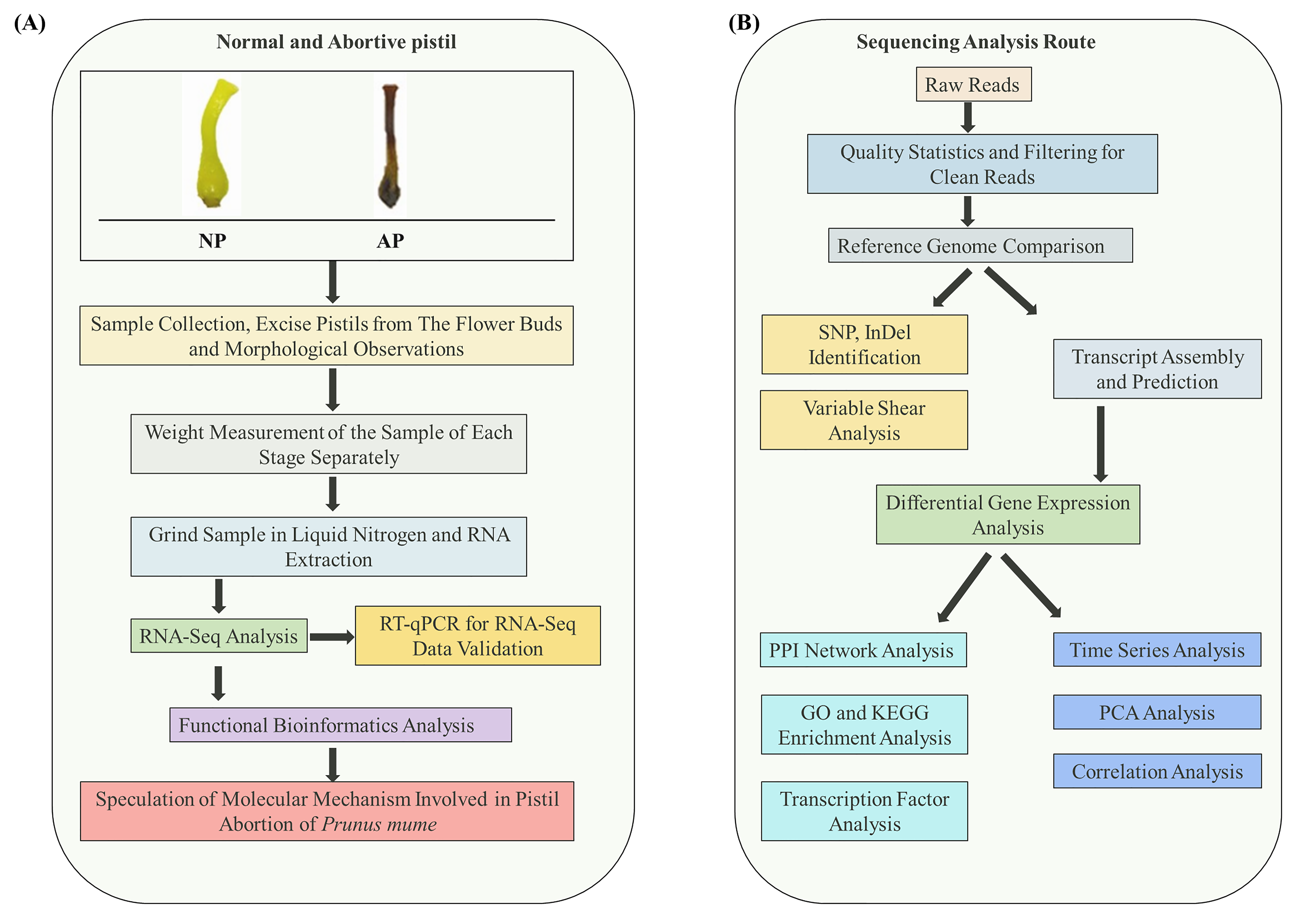

Supplement: Supplementary file 1 [file genes-11-01079-s001.zip › Supplementary materials/Supplementary Figures/Fig S1.png]

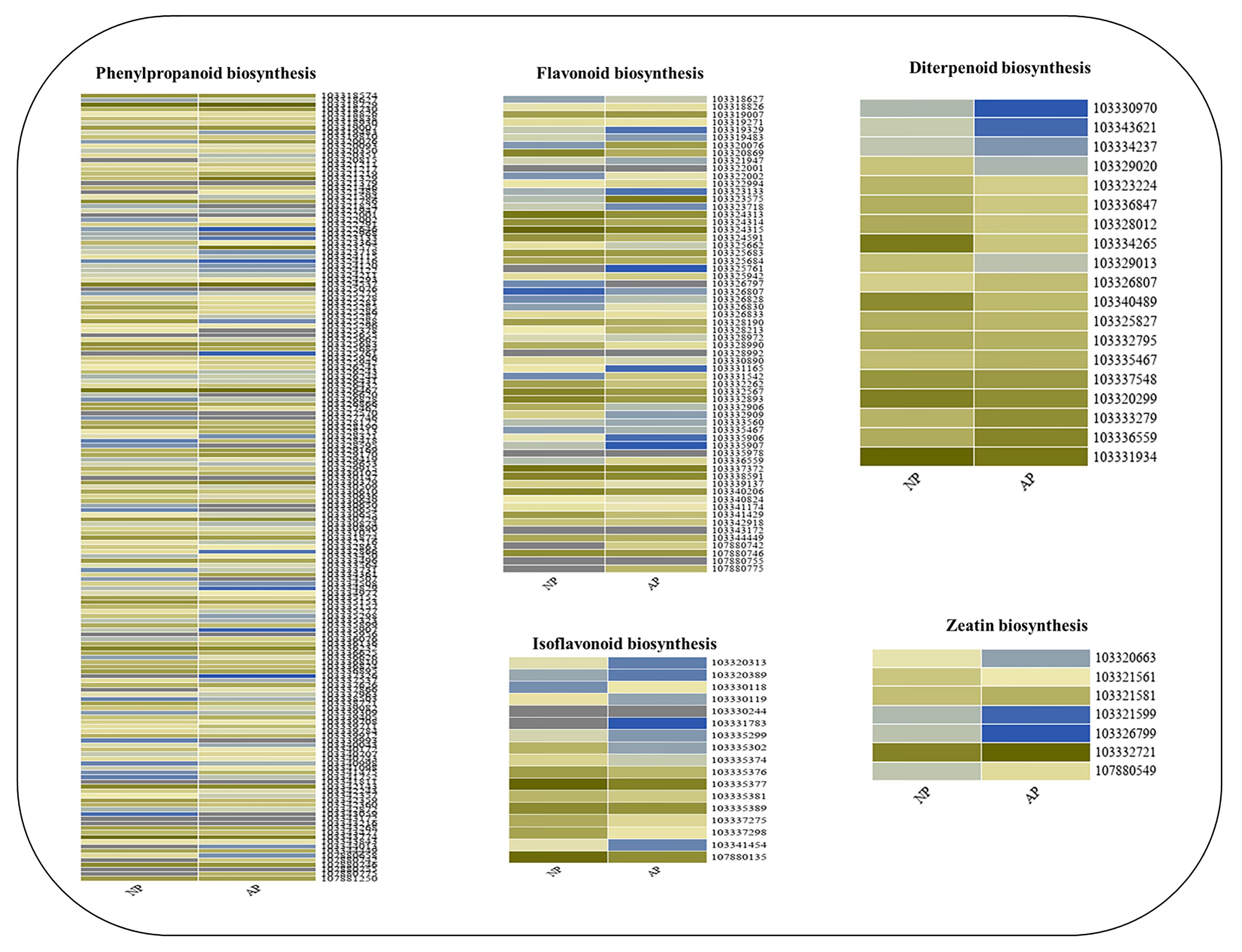

Supplement: Supplementary file 1 [file genes-11-01079-s001.zip › Supplementary materials/Supplementary Figures/Fig S2.png]
